# How to achieve low-carbon development in China: spatial spillover of education on carbon intensity

**DOI:** 10.3389/fpubh.2025.1567153

**Published:** 2026-01-30

**Authors:** Congying Ma, Hongchao Wu

**Affiliations:** 1 Department of Education, School of Educational Sciences, Lingnan Normal University, Zhanjiang, China; 2 School of Education, Beijing Institute of Technology, Beijing, China; 3 School of Education, South China Normal University, Guangzhou, China; 4 School of Education, Kashi University, Kashi, China

**Keywords:** education, carbon intensity, spatial Durbin model, spillover effect, influence mechanism

## Abstract

**Introduction:**

With the looming global warming crisis, the question of how to achieve carbon neutrality is becoming an important issue confronting many countries. Although the spatial studies have indicated that education can spatially reduce the carbon intensity, they have ignored the transmission mechanism and dynamic interrelationship between education and carbon intensity.

**Methods:**

Based on spatial economics theory, this study uses a spatial Durbin model and spatial vector autoregressive model to examine how education affects carbon intensity and how they relate to each other in China’s 30 provinces from 2000 to 2021.

**Results:**

The results show that education and the carbon intensity have positive global spatial autocorrelation. Education reduces the carbon intensity mainly through the spillover effect. The negative spillover effects in provinces with low carbon intensity are significantly greater than the direct effects are, whereas the spillover effects in provinces with high carbon intensity are negative but not significant. Education reduces the carbon intensity through green technology innovation and consumption structure upgrading. Furthermore, there is a dynamic interrelationship between education and carbon intensity. This study suggests that local governments should increase investment in education and focus on educational equity. They should also consider the spatial spillover effects of education when implementing tailored emission reduction policies based on local conditions. Additionally, educational institutions are encouraged to invest more in researching and promoting green technologies, as well as integrating low-carbon lifestyles into the education system.

**Discussion:**

The findings can help policymakers achieve sustainable development goals 4 and 13 and a net-zero future.

## 1 Introduction

Global economic growth, rapid industrialisation, and a sharp increase in energy consumption have contributed to the enormous increase in greenhouse gas emissions and spurred climate vulnerabilities (1). Climate change has hampered global socioeconomic and ecological environmental stability and thus has emerged as the greatest risk to humanity in the 21st century (2). Therefore, there is now a consensus among major economies worldwide on how to achieve carbon neutrality. Although the world economy will have to rely on natural resources (e.g., oil and gas) for energy production in the foreseeable future, governments and researchers worldwide have made efforts to achieve net zero emissions and limit ecological issues. Proper solutions have become highly relevant for implementing the Paris Agreement and 2030 global agenda.

According to recent reports, China has been the world’s largest emitter of greenhouse gases and the main source of growth in global carbon emissions (CEs), making its strategies pivotal to achieving the global carbon neutrality goals. In response, the Chinese government has proposed a series of low-carbon strategies (e.g., the 2030 carbon peak and 2060 carbon neutrality) (3, 4). Although some progress has been made, the carbon intensity (CI) in China is much greater than that in developed countries (4). Hence, CI reduction has become one of the most prevalent topics and a growing concern for stakeholders, governments and scholars. Moreover, an increasing number of studies have explored CI reduction solutions. However, these studies often focus on the role of economic variables, technological innovation, and institutional design in reducing the CI (5–7).

As sustainable development goal 4 (SDG 4) states, education (Edu) is important for the sustainable development of a nation (8). In recent years, the relationship between Edu and the CI has progressively emerged as a prominent topic in the field of environmental protection. Researchers have proven that Edu could be important in reducing the carbon intensity by enhancing environmental awareness and sustainable habits (9). For example, educated individuals prefer to adopt pollution-free technologies, pay carbon taxes, and adhere to environmental norms in industries, transportation, and households (10, 11). Thus, Edu will likely be a critical factor in the future achievement of a low-carbon society.

Against this background, the Chinese government is also aware of the green role of Edu and has been building a green and low-carbon development education system (12). For example, the Chinese government has integrated environmental education into the curriculum, developed green and low-carbon related professional disciplines and built green campuses. Although China achieved SDG 4 (quality education) in 2022, there are still some challenges, such as insufficient education levels, uneven regional education development and an unclear understanding of the low-carbon role of Edu, which strongly affect CI reduction (13). Hence, in-depth analysis of the effect of Edu on the CI in China can inform policymakers to develop carbon reduction policies and more effectively consider the impacts of Edu and the spatial effects of neighbouring regions, which can further promote investing more in Edu. They also help policymakers in countries worldwide develop carbon reduction policies to achieve the SDGs 4 and 13 towards a net-zero future and mitigate global warming. In particular, our findings may provide useful suggestions for countries experiencing the transformation of economic development patterns (e.g., from traditional to sustainable growth).

At present, there are still some research gaps. First, despite its importance, the effect of Edu on the CI is controversial in various countries (3, 14). Notably, China, as the world’s largest developing country and carbon emitter, is mainly responsible for global warming; it also faces immense pressure to balance its economic ambitions with sustainable practices. Thus, it is necessary to further estimate the role of Edu in reducing CI in China. Second, spatial economics theory suggests that educated individuals are mobile and will migrate between regions, which may impact the CI of local and surrounding areas (15). With increasing regional integration, the spillover effects of Edu and the CI have gradually increased. Although the existing spatial studies have discussed the spillover effect of Edu on the CI, which are limited to the verification of the relationship between Edu and the CI (16, 17), the transmission mechanism has not been described systematically. Finally, most of the spatial studies have not discussed the dynamic interrelationship between Edu and CI (18, 19).

To fill this gap, this study focuses on the following questions: What spillover effects and transmission mechanisms do Edu have on the CI? What is the dynamic interrelationship between Edu and CI? In response, on the basis of spatial economics theory, we analyse the impacts of Edu on the CI in China. First, we test the spatial autocorrelation of Edu and the CI. Additionally, we build a spatial Durbin model (SDM) to explore the spillover effects of Edu on the CI. We further we conduct heterogeneity tests of CI levels, followed by an impact mechanism analysis. Finally, we employ a spatial vector autoregression model (SpVAR) to explore the interrelationship between Edu and CI in a dynamic way.

This study makes three contributions. First, this study integrates spatial economics theory into SDG 4, estimating the role of education in sustainability. The results will serve as a foundation for incorporating educational spatial spillover into environmental policy frameworks. Second, this study estimates the spillover effect of Edu on the CI, followed by heterogeneous tests based on the SDM. These interesting findings expand upon the existing findings of spillover-free and spatial-autocorrelation-free and have practical significance for guiding the flow of talent and carrying out regional synergistic carbon reduction strategies tailored to local conditions. Then, carbon neutrality initiatives are closely related to both the technical and individual levels. Thus, we are not limited to verifying relationships and further revealing the mechanism of Edu’s influence on the CI from the perspectives of green technology innovation (GTI) and consumption structure upgrading (CSU), which empirically design paths to achieve carbon neutrality and expand on previous results. Finally, we explore the interrelationship between Edu and CI in a dynamic way, which support amenity theory and provide empirical evidence to illuminate the “black box” of their dynamic interrelationship.

The rest of the paper is organised as follows: Section 2 introduces the relevant literature review and theoretical hypotheses; Section 3 describes the variables and methodology; Section 4 reports the empirical results, followed by further analyses in Section 5; Section 6 tests the robustness of the SDM; Section 7 discusses the results, followed by the conclusions and recommendations in Section 8; and Section 9 provides limitations and future work.

## 2 Literature review and theoretical hypotheses

### 2.1 Literature review

Maintaining economic growth while reducing CEs is a challenge because CEs are associated with economic activities performed by humans. As endogenous growth theory affirms that educated individuals are important for a country to achieve the SDGs (20), many empirical studies validate how education could be associated with environmental degradation from different perspectives. Owing to differences in methods, sample data and study periods, three distinct categories of results are obtained.

#### 2.1.1 Direct effect of education on the carbon intensity

Some researchers have suggested that Edu mitigates CO_2_ emissions in OECD economies (21, 22), in China (16, 23–25). A positive role of Edu in reducing the carbon intensity has also been reported in China (3, 7). Similarly, Zheng et al. (26) reported that the mechanism driving the impact of the carbon intensity reduction targets centres on the level of education. Similar findings have been reported by Zafar et al. (27) and Ahmed et al. (28), who assessed the OECD economies and China and showed that education reduces the ecological footprint. Another study conducted by Gandhi et al. (29) revealed that physical meetings and continuing medical education reduces ecological footprint. Similarly, Zafar et al. (30) suggested a negative relationship between education and emissions for top remittance-receiving countries and China, respectively. One study inferred that human capital positively enhances Chinese ecological innovation in the long and short run (31). Lee et al. (32) also suggested that tertiary education helps decrease CEs per capita in countries with higher PGDP.

Conversely, numerous studies have shown that Edu positively impacts CEs (14, 33) or expands the ecological footprint in Pakistan (34). Other studies also revealed a significantly positive association between education and emissions for high-HDI countries (35), BRICS countries (36), and China (37). Similarly, Zhang et al. (38), for Indonesia and Sub-Saharan Africa, found that education expenditures contribute to deteriorating environmental quality. Similar findings have been reported by Sarwar et al. (39), who found that higher education is not useful for controlling CO_2_ emissions reduction process.

Furthermore, some studies have revealed that the effects of Edu on CEs exhibit time-stage heterogeneity (40), educational stage heterogeneity (41), and regional or national heterogeneity (19). Additionally, researchers have employed the traditional econometric models and argued that the direct effect of Edu on the CI shows an inverted N-shaped approach (42) or inverted U-shaped pattern (43). One study has also found that human capital has a stronger impact on environmental deficit at the higher quantiles (44). Çakar et al. (45) have shown that human capital decreases CEs in the low growth regime, whereas it increases in the high growth regime.

#### 2.1.2 Spillover effect of education on the sustainability variables

Few studies also have estimated the spillover effect of Edu on sustainability variables. For example, one study has shown that education investment can significantly reduce CEs through spillover effects (46). Another study reported that educational human capital has significantly positive direct effects but not significant spillover effects, which restricts the development of green total factor productivity (GTFP) (47). Vagnini et al. (18) used a spatial econometric model that illustrates that higher levels of education have negative direct and spillover effects on industrial CO2-eq emissions.

#### 2.1.3 Causality relationship between education and CEs

There is only a handful of papers that have dealt with the causality relationship between education and CEs. For example, Shafiullah et al. (48) detected a two-way causality between education and carbon emissions. Similar findings have been reported by Ketenci (49), who finds that the level of education and energy consumption are significant determinants of CEs and there is a two-way causality relationship between these variables.

In summary, these studies provide a research foundation, which shows that Edu can promote environmental sustainability or damage. However, several gaps still remain. First, the influence of Edu on the CI has failed to reach a consensus globally, necessitating further inquiries. Furthermore, despite the evolution of spatial econometrics, the corresponding research fails to estimate the spillover effect and impact mechanism of Edu on the CI. Finally, the existing studies have not discussed the dynamic interrelationship between Edu and CI.

Thus, to fill these gaps, this study analyses the impacts of Edu on the CI in China. We first construct a more accurate SDM model to estimate spatially lagged terms, which has a statistical advantage over the spatial error model (SEM) and spatial lag panel (SLM), which estimate spatially lagged terms, both the Edu and CI variables, and alleviates the endogeneity problem and estimate bias caused by ignoring spatial lag (15). This model also distinguishes between direct effects and spatial spillover effects, providing more refined policy implications. Employing this model, we analyse the spillover effect of Edu on the CI, followed by heterogeneity tests and transmission mechanism. Then, we build a SpVAR model, which combines the characteristics of traditional VAR models and spatial econometrics. Its core advantage is to capture the temporal dynamics and spatial dependence between variables at the same time, reveal the spatial interaction mechanism that is difficult to capture by traditional models, and provide a more comprehensive perspective for policy making and complex system analysis (15). Employing this model, we explore the dynamic interrelationship between Edu and CI.

### 2.2 Theoretical hypotheses

#### 2.2.1 Direct effect of education on the carbon intensity

In general, endogenous growth theory suggests that Edu can directly reduce the CI through green support and capital investment. On the one hand, education is a useful tool for increasing awareness of the spending patterns of economic agents (38), which can potentially awaken individuals’ environmental consciousness and develop social responsibility, enabling them to adopt environmentally friendly lifestyles and actively reduce the carbon intensity (41). For example, education has been consistently shown to increase environmental concern, willingness to pay for environmental protection, and support for environmental policy and products (50). On the other hand, education is a main supplier of the green workforce, effectively providing sufficient and high-quality human capital for green production to promote the green optimisation of socioeconomic activities (51). However, existing studies have indicated that Edu can increase CI (14, 33). Reasonable explanations are as follows: First, the conclusions can be explained by the differences in sample and methods, as well as endogeneity problems or missing variables. For example, static statistical methods ignore the spatial spillover of the variables, which lead to inaccurate results. Second, while rapid expansion of Edu may have negative consequences in the short-term (for example, in terms of energy consumption and pollutant emissions), it has a positive impact in the long-term. Because Edu promotes individual environmental awareness and lifestyles, which may take a long time to manifest the green value of Edu. Thus, Hypothesis 1 is proposed.

*Hypothesis 1*: Edu helps reduce the CI.

#### 2.2.2 Spillover effect of education on the carbon intensity

Spatial economics theory suggests that Edu has positive spatial interconnections due to talent mobility and knowledge spillover (15). With increasing regional integration and the development of spatial economics theory, this assumption is further extended to three spillover channels. First, educated individuals migrate across surrounding provinces, which accelerates the spillover of knowledge (52). Second, educational patterns affect neighbouring provinces through demonstrations and competition (53). Through these channels, a province’s CI depends on the educational human capital (EHC) in the local province and on the speed and quality of the EHC overflow from neighbouring provinces. Thus, Hypothesis 2 is proposed.

*Hypothesis 2*: Edu has spillover effects on the CI in neighbouring provinces.

#### 2.2.3 Impact path of education on the carbon intensity

Endogenous growth theory suggests that human capital is the source of GTI, which contributes to the occurrence, introduction and absorption of green innovation (54). In addition, educated individuals’ demand for green products will be stronger, which will indirectly push firms to accelerate GTI. Finally, the educated public can experience behaviours that damage the environment and are not conducive to carbon neutrality in a variety of ways, which can promote GTI. Ecological modernisation theory also suggests that GTI is a cornerstone of climate change mitigation at the global scale (55) and that the powerful productivity caused by GTI substantially accelerates the decarbonisation of the economy. Thus, Hypothesis 3a is proposed.

*Hypothesis 3a*: Edu reduces the CI via GTI.

The education level of individuals determines people’s cognitive level of low-carbon development and consumption (56). Furthermore, eductaion can reshape people’s low-carbon consumption habits. For example, educational curricula and programs that promote healthy eating habits include meaningful reflections on resource exploitation associated with food production, distribution, consumption, and disposal. Education also reshapes consumption habits by fostering critical awareness, and aligning spending with values (health, sustainability, culture). Furthermore, education upgrade their consumption structure, and increase their consumption of less carbon-intensive goods and services (57), such as saving energy, recycling, saving water, preferring eco-labelled food, and eco-labelled appliances, thus reducing the carbon intensity in the residential sector. Some studies have underlined the role of education in encouraging the adoption of more sustainable and healthier food consumption. Education background positively impacts the low-carbon living behaviour and low-carbon purchase behaviour. Individual consumption behaviours have been shown to be an influential indirect force in emissions abatement (58). Thus, Hypothesis 3b is proposed.

*Hypothesis 3b*: Edu reduces the CI via the CSU.

#### 2.2.4 Dynamic interrelationship between education and carbon intensity

According to Hypothesis 2, it is known that Edu has spatial effects on the CI. Amenity theory also suggests that the superior natural environment affects people’s quality of life and attracts talent to gather. Improvements in the CI, which is in environmental indicators, can also be interpreted as an indicator of social welfare. This situation can also affect many variables, such as education in the country. Therefore, it can be interpreted as the improvements in environmental indicators (e.g., CI) in neighbouring provinces and years may affect education policies in the short term (59). With the development of the economy, this impact may become increasingly significant. Furthermore, current values of education correlate strongly with past values of carbon emissions, and vice versa (49). Thus, Hypothesis 4 is proposed.

*Hypothesis 4*: There is a dynamic interrelationship between Edu and CI.

**Figure 1:**
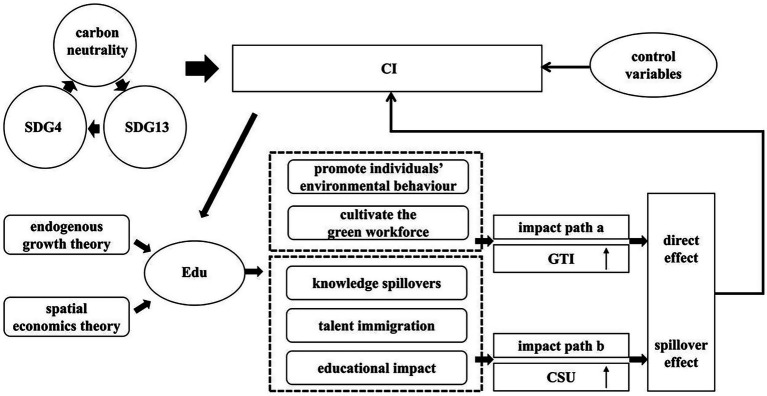
Theoretical framework.

On the basis of the above analysis, the theoretical framework of this study is proposed in Figure 1.

## 3 Materials and methods

### 3.1 Materials

#### 3.1.1 Dependent variable

The dependent variable is the CI. The CI is calculated by the regional total CO_2_ emissions/regional GDP, which is an effective indicator for countries worldwide to measure the balanced development of economic and ecological environmental protection (60). Compared with total carbon emissions or per capita carbon emissions, the CI has been proven to be more practical as a measure of energy intensity and as a guide for allocating responsibility for reducing emissions (61, 62).

#### 3.1.2 Explanatory variable

The explanatory variable is education. The measurement methods of education include the education stock method, cost–benefit method, and cumulative investment method. In this study, education is calculated by the education stock method, which mainly calculates the aggregate number of years of education per capita, which refers to the accumulation of knowledge and skills acquired through investment in education by the labor force. It can comprehensively reflect the average education level of the labor force (16). Equation 1 is set as follows:


$$\mathrm{Ed}u_{\mathrm{it}}=\sum_{i=1}^{n=6}p_{\mathrm{it}}y_{\mathrm{it}}/P_{\mathrm{it}}$$ (1)

where n is the educational level; y_it_ represents the education years corresponding to i level of education at time t; p_it_ represents the total population receiving i level of education at time t; P_it_ represents the total population aged six and over. At present, there are mainly 7 educational levels in China, including primary school, junior high school, high school/secondary vocational school, junior college/higher vocational colleges, college, master’s, and doctoral students. However, the number of educated people at multiple levels, such as vocational school and doctoral students, is seriously lacking at the statistical level. In response to this issue, this article simplifies the education hierarchy and divides education into six levels, namely, primary school, junior high school, high school, junior college, college education, and master, and the education years received at each level are defined as 6 years, 9 years, 12 years, 15 years, 16 years, and 19 years, respectively. Thus, $y_{\mathrm{it}}=6,9,12,15,16,19$ .

#### 3.1.3 Mechanism variables

1.GTI. Green technology refers to a combination of emission-reduction technology, energy-efficiency technology, and renewable energy technology. Stronger green technology innovation capabilities are conducive to expanding the use of renewable energy and decreasing energy consumption and CO_2_ during production (46). GTI is represented by the number of green invention patents granted.

2.CSU: In the process of upgrading the consumption structure, educated consumers pay more attention to enjoyment and development consumption, and this type of consumption helps to transform resource and environmental utilisation patterns and promote green economic development (63). Equation 2 is set as follows:


$$\mathrm{CS}U_{t}=\sum_{i=1}^{2}\frac{P_{\mathrm{it}}}{P_{t}}(\frac{\mathrm{Ju}n_{\mathrm{it}}}{C_{\mathrm{it}}}+\frac{\text{In}t_{\mathrm{it}}}{C_{\mathrm{it}}}\times 2+\frac{\mathrm{Se}n_{\mathrm{it}}}{C_{\mathrm{it}}}\times 3)$$ (2)

where Jun_it_, Int_it_ and Sen_it_ represent primary consumption, intermediate consumption and senior consumption, respectively. The weights of primary consumption, intermediate consumption and senior consumption are 1/6, 2/6 and 3/6, respectively; C_it_ and P_it_ represent the total consumption and total population in rural or urban areas at time t, respectively; and i is used to distinguish urban and rural areas; P_t_ represents the total population in rural and urban areas at time t.

#### 3.1.4 Control variables

According to the STIRPAT model (64), the CI is affected by many factors (e.g., population, affluence, and technology). In this study, we further expand the model and adopt the following control variables: foreign direct investment (FDI), financial development (FD), information and communication technology (ICT), population structure (Pop), and economic development (PGDP) (Table 1).

**Table 1:** Control variable selection and symbols.

Variable	Definition	Symbol
Foreign direct investment	Amount of foreign direct investment/provincial GDP	FDI
Financial development	(Deposits and loans)/provincial GDP	FD
Information and communication technology	Postal and telecommunications business volume/provincial GDP	ICT
Population structure	Child-age dependency rate/older adult dependency rate	Pop
Economic development	Provincial GDP per capita	PGDP

1.FDI: According to the “pollution halo” theory, the more open a region is, the greater the likelihood that it will bring advanced management experience and clean technology, which will help reduce the CI. However, the “pollution sanctuary” hypothesis suggests that, due to the more stringent environmental regulations in developed countries, FDI often leads to the transfer of energy-intensive and high-polluting industries to China, leading to an increase in pollution emissions (19).

2.FD: FD is the catalyst for economic activities and interacts with environmental factors (65). It supports R&D activities related to low-carbon technologies, helps the home country adopt existing low-carbon technologies from other countries, or confers superior financial services for environmentally friendly programs at lower costs.

3.PGDP: The EKC demonstrates that the relationship between economic growth and the CI is complex because when a country starts to grow, economic growth is also accompanied by increasing combustion of fossil energy, thus increasing CO_2_ emissions. However, in the later stages, due to the use of sophisticated technologies, modern production methods, and the demand for clean and green products, the environmental quality starts to improve (5).

4.POP: Shifts in age can reduce CO_2_. For example, a higher older adult dependency rate makes social labour factors scarce, which helps reduce the degree of carbonisation of the industrial structure (66).

5.INF: INF has significant potential for reducing emissions by promoting technological innovation, optimising the energy structure, and promoting clean energy enterprises. However, the development and construction of IINF infrastructure may also consume nonrenewable energy, which could adversely affect carbon emissions (39).

#### 3.1.5 Sample and data sources

This study uses panel data from China’s 30 provinces from 2000 to 2021 (Tibet, Hong Kong, Macao and Taiwan are excluded because of a lack of data). The CI data come from the MEIC database, which uses the emission factor method to calculate carbon emissions on the basis of subsectoral energy consumption (67). All other data sources include the China Statistical Yearbook, the China Population and Employment Statistics Yearbook, and the Chinese Research Data Services Platform. All the research data are estimated at the 2000 price level, and the CI, Edu, PGDP and GTI are in logarithmic form.

Table 2 displays the descriptive statistics of the variables. This result shows that the CI shows a downwards trend and Edu shows an upwards trend, suggesting that the local government is paying increasing attention to achieving carbon neutrality and developing education. Furthermore, the CI and Edu exhibit regional differences. The CI values are west>middle>east, and the Edu values are east>middle>west, suggesting that the CI and Edu values are closely related to the local government’s PDGP. For example, East China is the richest, with the lowest CI and the highest education level.

**Table 2:** Descriptive statistics.

Variable	Obs	Mean	Std. dev.	Min	Max
lnCI	660	−8.552	0.678	−10.722	−6.955
lnEdu	660	2.151	0.131	1.693	2.548
lnGTI	660	4.822	1.926	0	9.114
CSU	660	1.046	0.097	0.757	1.316
FDI	660	0.024	0.021	0.000	0.146
FD	660	2.991	1.079	1.413	7.578
ICT	660	0.062	0.041	0.014	0.290
Pop	660	2.045	0.967	0.536	6.400
lnPGDP	660	9.185	0.522	7.887	10.781

Table 3 shows that all variance inflation factors (VIFs) are lower than the critical value of 10, with no multicollinearity problems.

**Table 3:** Results of the multicollinearity test.

Variable	VIF
lnEdu	4.400
lnGTI	4.130
CSU	2.000
FDI	1.700
FD	2.140
ICT	1.190
Pop	3.040
lnPGDP	4.040

Theoretically, the dependent and independent variables are likely time persistent; therefore, it is necessary to conduct a stationarity test for the variables to avoid possible spurious regression. We use the panel unit root test, presenting the results in Table 4, confirming that the variables are stationary.

**Table 4:** Global Moran’s I index of Edu and the CI.

Year	Edu	CI	Year	Edu	CI
2000	0.424^***^	0.186^**^	2011	0.374^***^	0.214^***^
2001	0.412^***^	0.203^**^	2012	0.394^***^	0.233^***^
2002	0.412^***^	0.207^***^	2013	0.408^***^	0.226*^**^
2003	0.341^***^	0.181^**^	2014	0.384^***^	0.232^***^
2004	0.407^***^	0.189^**^	2015	0.343^***^	0.240^***^
2005	0.385^***^	0.202^**^	2016	0.354^***^	0.246^***^
2006	0.385^***^	0.179^**^	2017	0.344^***^	0.214^***^
2007	0.358^***^	0.189^**^	2018	0.358^***^	0.220^***^
2008	0.371^***^	0.230^***^	2019	0.370^***^	0.224^***^
2009	0.380^***^	0.219^***^	2020	0.325^***^	0.222^***^
2010	0.376^***^	0.232^***^	2021	0.340^***^	0.219^***^

*p < 10%, **p < 5%, and ***p < 1%.

### 3.2 Methods

#### 3.2.1 Spatial econometric model

Researchers have shown that Edu and the CI are spatially dependent (16, 68). However, most studies that have investigated the CI and Edu nexus are based on the independence assumption, which may lead to biased estimation results (21, 22). Thus, it is necessary to further estimate the spillover effects of Edu on the CI.

First, the spatial correlation between Edu and the CI is tested through the global Moran’s I index. This is calculated as follows:


$$I=\frac{n\sum_{i=1}^{n}\sum_{j=1}^{n}W_{\mathrm{ij}}(X_{i}-\bar{X})(X_{j}-\bar{X})}{\sum_{i=1}^{n}\sum_{j=1}^{n}W_{\mathrm{ij}}\sum_{i=1}^{n}{\left(X_{i}-\bar{X}\right)}^{2}}$$ (3)

where X_i_ denotes the observed value of province i, i.e., Edu and the CI; n is 30; and the range of Moran’s I is [−1, 1]. A Moran’s I value close to −1 or 1 indicates that the level of global spatial autocorrelation is high (15).

In Equation 3, W_ij_ denotes the geographic distance spatial weight matrix, which is expressed in Equation 4:


$$W_{\mathrm{ij}}=\{\begin{array}{l} \frac{1}{{d_{\mathrm{ij}}}^{2}},i\neq j\\ 0,i=j \end{array}$$ (4)

where d_ij_ denotes the geographic distance between the provincial capital cities of province i and province j, which is calculated using latitude and longitude data. Assuming that the latitude of the capital city of province i is *β*_1_ and the longitude is α_1_, and the latitude of the capital city of province j is β_2_ and the longitude is α_2_, R is the radius of the Earth, $d_{\mathrm{ij}}=R*\mathrm{arc}\;\cos[\cos\beta_{1}\cos\beta_{2}\cos(\alpha_{1}-\alpha_{2})+\sin\beta_{1}\sin\beta_{2}]$ . If province i is closer to province j, W_ij_ will increase (15).

Then, on the basis of the regression on the population, affluence, and technology (STIRPAT) framework (61), we construct a more accurate SDM model to explore the spillover effects of Edu on the CI. It can be expressed as follows:


$$\begin{array}{l} {\text{lnCI}}_{\mathrm{it}}=\rho \sum_{j=1}^{n}W_{\mathrm{ij}}\mathrm{lnC}I_{\mathrm{it}}+\beta_{1}{\text{lnEdu}}_{\mathrm{it}}+\beta_{2}\text{lnContro}l_{\mathrm{it}}\\ +\sum_{j=1}^{n}\theta_{1}W_{\mathrm{ij}}{\text{lnEdu}}_{\mathrm{it}}+\sum_{j=1}^{n}\theta_{2}W_{\mathrm{ij}}\text{lnContro}l_{\mathrm{it}}+u_{i}+\nu_{t}+\varepsilon_{\mathrm{it}} \end{array}$$ (5)

where *ρ* and *θ* denote the spatial lagged coefficients of the variables; β denotes the regression coefficients of the variables; and the remaining variables are set as above (15).

However, the SDM cannot directly estimate spillover effects. Thus, the partial differential method is finally employed to calculate the direct and spillover effects of Edu on the CI (69), which are expressed in Equations 6–[Disp-formula EQ7] 8.


$$\text{Direct effects}={\left[{\left(I_{N}-\mathrm{\rho W}\right)}^{-1}(\beta_{k}I_{N}+\theta_{k}W)\right]}^{\bar{d}}$$ (6)

$$\text{Spillover effects}={\left[{\left(I_{N}-\mathrm{\rho W}\right)}^{-1}(\beta_{k}I_{N}+\theta_{k}W)\right]}^{\bar{\text{rsum}}}$$ (7)

$$\begin{array}{l} \text{Total effects}={\left[{\left(I_{N}-\mathrm{\rho W}\right)}^{-1}(\beta_{k}I_{N}+\theta_{k}W)\right]}^{\bar{d}}\\ +{\left[{\left(I_{N}-\mathrm{\rho W}\right)}^{-1}(\beta_{k}I_{N}+\theta_{k}W)\right]}^{\bar{\text{rsum}}} \end{array}$$ (8)

where I_N_ is an identity matrix, k represents the k-th variables, and ^−^d and^−^rsum represent two operators that can be used to estimate both the mean diagonal element and the mean row sum of the nondiagonal elements of the matrix.

#### 3.2.2 Interaction term model

To further explore the transmission mechanism through which Edu affects the CI, the SDM is taken as an example to build an interaction term model to estimate such transmission channels (e.g., GTI and CSU). On the basis of Equation 5, the models are expressed in Equations 9, 10:


$$\begin{array}{l} {\text{lnMec}}_{\mathrm{it}}=\rho \sum_{j=1}^{n}W_{\mathrm{ij}}\ln\mathrm{Me}c_{\mathrm{it}}+\beta_{1}\ln\mathrm{Ed}u_{\mathrm{it}}+\beta_{2}\text{Contro}l_{\mathrm{it}}\\ +\theta_{1}\sum_{j=1}^{n}W_{\mathrm{ij}}\ln\mathrm{Ed}u_{\mathrm{it}}+\theta_{2}\sum_{j=1}^{n}W_{\mathrm{ij}}\text{Contro}l_{\mathrm{it}}+u_{i}+\nu_{t}+\varepsilon_{\mathrm{it}} \end{array}$$ (9)

$$\begin{array}{l} {\text{lnCI}}_{\mathrm{it}}=\rho \sum_{j=1}^{n}W_{\mathrm{ij}}\ln CI_{\mathrm{it}}+\beta_{1}{\text{lnEdu}}_{\mathrm{it}}+\beta_{2}{\text{lnEdu}}_{\mathrm{it}}\times l{\text{nMec}}_{\mathrm{it}}\\ +\beta_{3}\text{Contro}l_{\mathrm{it}}+\theta_{1}\sum_{j=1}^{n}W_{\mathrm{ij}}\ln\mathrm{Ed}u_{\mathrm{it}}+\theta_{2}\sum_{j=1}^{n}W_{\mathrm{ij}}{\text{lnEdu}}_{\mathrm{it}}\times l{\text{nMec}}_{\mathrm{it}}\\ +\theta_{3}\sum_{j=1}^{n}W_{\mathrm{ij}}\text{Contro}l_{\mathrm{it}}+u_{i}+\nu_{t}+\varepsilon_{\mathrm{it}} \end{array}$$ (10)

where Mec is the mechanism variable, i.e., GTI and CSU. LnEdu_it_ × LnMec_it_ denotes the interaction term between Edu and Mec. In Equation 11, β_2_ is the coefficient of the interaction term and denotes the degree to which Edu adjusts the direct effect of Mec on the CI, and ϴ_2_ denotes the degree to which the spillover effect is regulated (70). The other variables are set as before.

#### 3.2.3 Spatital vector autoregressive model

Researchers have shown that Edu reduces CI. Thus, the interrelationship between Edu and CI is tested through a SpVAR in a dynamic way, which introduces two dimensions of spatial and temporal lag. To address endogeneity issues, we adopt the method of generalized matrix estimation. Firstly, remove fixed effects from the differential equation and select lagged explanatory variables as instrumental variables for the corresponding variables in the differential equation (71). It can be expressed as follows:


$$(\Delta Y_{t})\prime =[(\Delta Y_{t-1}\prime ),(\Delta Y_{t-1}^{*})\prime ][\prod_{2}^{\prime }\prod 1\prime ]+(\Delta \upsilon_{t})\prime$$ (11)

where Y is the column vector of NK × 1; ∏_1_ = M × I_N_ × β_kj_; ∏_2_ = M × I_N_ × λ_ki;_ ∆υ_t_ = M∆ε_t_; M = (I_Nk_-I_N_ × α_ki_)^−1^; ∆Y_t_’ represents the transpose matrix of the first-order difference matrix; t is the time series; j is the lag order; n is the spatial influencing factors; k is the k-th variables; I_N_ and M is an identity matrix; β_kj_, λ_ki_, and α_ki_ are the coefficients to be estimated; ε_t_ is the residual disturbance term; and Y_t-1_ is the spatial lag term of Y_t,_ andY_t-1_^*^ is the spatial lag term of Y_t-1_.

Then, we choose a set of lagged explanatory variables as instrumental variables:

$\left[\begin{array}{l} Y_{t-2},Y_{t-3},\cdots ,Y_{1}\\ Y_{t-2}^{*},Y_{t-3}^{*},\cdots ,Y_{1}^{*} \end{array}\right]$ is the instrumental variable of $\left[\begin{array}{l} Y_{t-1},Y_{t-2}\\ Y_{t-1}^{*},Y_{t-2}^{*} \end{array}\right]$ ; $\left[\begin{array}{l} \left(Y_{t-2}‐Y_{t-3}),(Y_{t-3}‐Y_{t-4}),\cdots (Y_{2}‐Y_{1}\right)\\ \left(Y_{t-2}^{*}‐Y_{t-3}^{*}),(Y_{t-3}^{*}‐Y_{t-4}^{*}),\cdots (Y_{2}^{*}‐Y_{1}^{*}\right) \end{array}\right]$ is the instrumental variable of $\left[\begin{array}{l} Y_{t-1}\\ Y_{t-1}^{*} \end{array}\right]$ .

## 4 Results

### 4.1 Spatial autocorrelation test

Table 5 shows that all the global Moran’s I values of Edu and the CI are significantly positive. The results reveal that the two variables have positive spatial autocorrelation, which is affected by geographic neighbours; that is, provinces with low Edu and CI are geographic neighbours, as are those with high Edu and CI.

**Table 5:** Test results of model selection.

Test	Value of a statistic
LM (err)	184.627^***^
Robust LM (err)	193.846^***^
LM (lag)	8.028^***^
Robust LM (lag)	17.247^***^
LR (err)	23.19^***^
LR (lag)	19.50^***^
Hausman test	12.82^**^
Wald test (err)	8.15^***^
Wald test (lag)	6.96^***^
LR (spatial fixed effects)	74.47^***^
LR (time fixed effects)	1112.61^***^

^*^p < 10%, ^**^p < 5%, and ^***^p < 1%.

Specifically, the global Moran’s I values for Edu present an overall downwards trend, which means that the spatial autocorrelation of Edu has been gradually decreasing. However, the CI shows a gradual upwards trend, indicating that the spatial correlation of the CI weakened from 2000 to 2021. These reasonable explanations are due to the demonstration and imitation effects among local government policies, which affect the spatial autocorrelation of Edu and the CI.

### 4.2 Model selection

According to the results of the spatial autocorrelation test, Edu and the CI exhibited spillover effects. Thus, the spatial econometric model is appropriate for this empirical investigation. On the basis of the conclusions of LeSage and Pace (69) and Elhorst (72), first, the Lagrange multiplier (LM) and robust LM tests indicate that the SDM is suitable. The likelihood ratio (LR) and Wald tests also show that the SDM could not be degraded to the SEM or SLM. Finally, Hausman tests and the joint significance likelihood ratio (LR) test are adopted to affirm that the SDM with double fixed effects is the most reasonable for regression. All the test results are shown in Table 6.

**Table 6:** Results of the SDM.

Variable	Main	Wx
lnEdu	−1.001^***^	−1.832^***^
	(0.273)	(0.695)
FDI	−0.931^*^	1.038
	(0.498)	(1.215)
FD	0.026	−0.018
	(0.025)	(0.056)
ICT	0.428	0.800
	(0.514)	(1.023)
Pop	−0.157^***^	−0.199^***^
	(0.028)	(0.060)
lnPGDP	−0.717^***^	0.173
	(0.075)	(0.168)
*ρ*	0.156^**^	0.156^**^
	(0.074)	(0.074)
*N*	660	660
*R* ^2^	0.595	0.280
Log-L	334.342	334.342

*p < 10%, **p < 5%, and ***p < 1%.

### 4.3 Basic regression

On the basis of spatial economics theory, an SDM is built for the whole country. The regression results are presented in Table 7.

**Table 7:** Spatial effect decomposition of SDM.

Variable	Direct	Indirect	Total
lnEdu	−1.047^***^	−2.324^***^	−3.371^***^
	(0.281)	(0.891)	(0.963)
FDI	−0.926^*^	1.003	0.077
	(0.488)	(1.366)	(1.568)
FD	0.028	−0.014	0.015
	(0.023)	(0.061)	(0.059)
ICT	0.443	1.044	1.487
	(0.483)	(1.123)	(0.976)
Pop	−0.163^***^	−0.260^***^	−0.423^***^
	(0.026)	(0.067)	(0.060)
lnPGDP	−0.710^***^	0.086	−0.623^***^
	(0.075)	(0.181)	(0.194)
*ρ*	0.156^**^	0.156^**^	0.156^**^
	(0.074)	(0.074)	(0.074)
*N*	660	660	660
*R* ^2^	0.280	0.280	0.280
Log-L	334.342	334.342	334.342

*p < 10%, **p < 5%, and ***p < 1%.

*ρ* is statistically significant at the 5% level, indicating that the CI has significant spatial correlations, which aligns with the spatial autocorrelation test results. A province’s increase in the CI by one unit can increase the CI in neighbouring provinces due to positive spillover effects. This finding shows that interprovincial spatial interaction is a key factor in reducing the CI.

The results show that the significant coefficient of Edu on the CI is −1.001, indicating that improvements in Edu would reduce the CI in China and increase environmental well-being. Thus, Hypothesis 1 is confirmed. This is plausible because as the number of years of education increases, it leads to green practices that assist in the transition to sustainable development models, for example, improving environmental awareness, following environmental policies, and adopting sustainable lifestyles (73). Therefore, improving Edu could act as a catalyst for sustainable development goals and reduce the reliance on replenishing natural resources.

The results also show that FDI, Pop and PGDP positively impact the reduction in the CI. (a) The advanced technology and better management practices introduced by FDI could improve industry efficiency in China and decrease the CI. This finding indicates that the technical effect of openness is greater than the structural effect, providing evidence for the pollution halo hypothesis (19). (b) With the increase in the child-age dependency rate and decrease in the older adult dependency rate, the energy consumption scale and consumption levels are decreasing (74, 75), and the consumption tendency is gradually focused on the high-tech technology industry and consumer service sectors, decreasing the share of energy-dependent industries and thus curbing the growth of the CI. (c) Consumers with higher per capita income demand consume more green products (e.g., low-energy-intensity household appliances). Therefore, this result also reveals that the Chinese economy is moving towards low-carbon development, which provides further evidence for the EKC (5).

### 4.4 Spillover effect analysis

Furthermore, the partial differential method is employed to decompose the impact of Edu on the CI into direct and spillover effects. The regression results are presented in Table 8.

**Table 8:** Results of the heterogeneity test.

Effect	(1)	(2)
	Low CI	High CI
Direct	−1.074^***^ (0.410)	−0.988^**^ (0.385)
Spillover	−2.719^***^ (0.920)	−0.719 (1.001)
Total	−3.794^***^ (1.057)	−1.707 (1.157)
Control variables	YES	YES
*ρ*	0.089 (0.076)	0.050 (0.095)
*N*	330	330
*R* ^2^	0.874	0.011
Log-L	200.514	187.522

*p < 10%, **p < 5%, and ***p < 1%.

The results show the significant direct, spillover and total effects of Edu on the CI, with regression coefficients of −1.047, −2.324 and −3.371, respectively, revealing that Edu not only reduced the CI in local provinces but also had negative spillover effects on neighbouring provinces. Thus, Hypothesis 2 is supported. Notably, the spillovers are larger than the direct effects are, revealing that spillover effects are the main channel through which Edu reduces the CI. The possible explanations are that educated individuals accept the concept of low-carbon development with both demonstration and peer effects (16). Furthermore, the human capital spillover also greatly promotes the spread of environmental awareness and increases the use of green production and consumption models across society.

### 4.5 Heterogeneity test

Different CI levels in provinces could result in disparities in the effect of Edu on the CI. Therefore, we sort the 30 provinces from high to low based on the average annual CI from 2000 to 2021. The top 15 provinces are set as high CI areas, and the remaining 15 provinces are set as low CI areas. The results are shown in Table 9.

**Table 9:** Results of intermediary effects.

Variable	GTI		CI		CSU		CI	
	Direct	Spillover	Direct	Spillover	Direct	Spillover	Direct	Spillover
lnEdu	2.791^***^ (0.606)	6.064^***^ (2.017)						
lnEdu × lnGTI			−0.387^***^ (0.039)	−0.162^**^ (0.091)				
LnEdu					0.124^**^ (0.059)	0.459^**^ (0.221)		
lnEdu×lnCSU							−3.234^***^ (0.676)	4.415^***^ (1.715)
Control variables	YES	YES	YES	YES	YES	YES	YES	YES
*ρ*	0.258^***^ (0.062)	0.258^***^ (0.062)	0.056^***^ (0.051)	0.056^***^ (0.051)	0.345^***^ (0.056)	0.345^***^ (0.056)	0.209^***^ (0.074)	0.209^***^ (0.074)
*N*	660	660	660	660	660	660	660	660
*R* ^2^	0.072	0.072	0.868	0.868	0.659	0.659	0.653	0.653
Log-L	164.896	164.896	384.052	384.052	1383.958	1383.958	353.279	353.279

*p < 10%, **p < 5%, and ***p < 1%.

Column (1) indicates that the significant regression coefficients of the direct, spillover and total effects of Edu on the CI are −1.074, −2.719 and −3.794, respectively, revealing that Edu significantly negatively impacts the CI in those provinces where the CI is low. The findings also show that spatial spillover is the main channel for reducing the CI in these provinces. While Column (2) shows that the direct effect of the significant regression coefficient of Edu on the CI is −0.988, the negative direct spillover and total effects are not significant when the CI are high. The findings indicate that the spatial spillover advantages of education in emission reduction are not significant and that educated individuals do not fully function in the application and diffusion of the green production mode and consumption model.

The possible reasons are as follows: Low CI areas are mainly in developed regions (e.g., the east), characterised by a high proportion of tertiary industry. However, high CI areas are mainly in developing regions (e.g., the central and western regions), where the economy has not entered the stage of sustainable development and where there are lower levels of human capital stock. Moreover, educated individuals are usually associated with less green technology and more environmental pollution (76), so they cannot spread the ideas of environmental protection through demonstration effects and migration behaviour. Furthermore, the pursuit of economic growth causes labourers to use more energy and produce more pollution, and the “rebound effect” greatly inhibits the role of Edu in reducing the CI in neighbouring areas.

### 4.6 Mechanism analysis

We further explore the transmission mechanisms through which Edu affects the CI via the interaction term model for more policy insights from the perspectives of GTI and CSU. lnEdu × lnGTI and lnEdu × lnCSU are centrally processed to avoid the statistical bias caused by multicollinearity. The results are shown in Table 10.

**Table 10:** Panel unit root tests.

Variable	ADF–Fisher statistic	LLC statistic
LnCI	75.608^*^	−6.501^**^
LnEdu	193.835^*^	−10.387^***^

*p < 10%, **p < 5%, and ***p < 1%.

#### 4.6.1 Green technology innovation

The significant direct and spillover effects of lnEdu on lnGTI are positive, and lnEdu × lnGTI on lnCI are negative, suggesting that Edu reduces the CI by regulating GTI. Thus, Hypothesis 3a is supported. This conclusion also supports the results of Sun (68). Specifically, increasing in Edu can accumulate innovative human capital and promote green technology progress. Moreover, educated individuals can improve their use of clean energy and green innovation willingness (16), which can help reduce energy consumption and the CI. In this study, we refine the impact path of GTI and more accurately grasp the impact mechanism of GTI.

#### 4.6.2 Consumption structure upgrading

The significant direct and spillover effects of lnEdu on lnCSU are positive, and the significant direct effects of lnEdu × lnCSU on lnCI on lnCI are negative, suggesting that Edu can synergistically reduce the CI by regulating CSU. Thus, Hypothesis 3b is supported. Specifically, education promotes consumption structure upgrading by improving individual environmental awareness to practice a low-carbon lifestyle. Some products that represent consumption upgrades often have low energy consumption and low pollution, which could reduce the CI. The findings support the evidence that education effectively promotes individuals’ environmental behaviour while empirically extending consumption structure upgrading as an important mechanism for the impact of Edu on the CI (63).

### 4.7 Further analysis: dynamic interrelationship analysis

We finally explore the interrelationship between Edu and CI in a dynamic way. Table 4 show the variables are stationary, which is suitable for further study. Thus, a SpVAR is built for the dynamic interrelationship analysis. The results are shown in Table 11.

**Table 11:** Results of the SpVAR.

Variable	LnEdu	LnCI
LnEdu (−1)	4.608 (2.960)	−5.806 (8.556)
LnCI (−1)	−1.109 (0.993)	2.988 (2.315)
LnEdu^*^ (−1)	−4.486 (3.056)	−6.273 (9.054)
LnCI^*^ (−1)	1.236 (1.101)	−2.302 (2.531)

*p < 10%, **p < 5%, and ***p < 1%.

The results indicate that (a) the effect of Edu (−1) and Edu* (−1) on CI is positive, which shows Edu has reduced the current CI in both temporal and spatial lag terms. This further confirms Hypothesis 1. (b) The effect of Edu (−1) on Edu is positive, which shows the positive lag effect of education on itself and also indicates cumulative human capital development. Higher past education levels (Edu-1) may promote institutional investment in schools. But the effect of Edu* (−1) on Edu is negative, which shows that there is competition among provinces in the development of education. (c) The effect of CI (−1) on CI is positive, which shows industries, infrastructure, and policies associated with high emissions will persist over time. But the effect of CI* (−1) on CI is negative, which shows there are pollution haven effects and races to the bottom in the development of CI between provinces. (d) The effect of CI (−1) on Edu is negative, but the effect of CI (−1) on Edu is positive, which shows that temporal lag CI has reduced the Edu, while the increase in CI in neighboring provinces has improved the local Edu. Thus, Hypothesis 4 is supported. One reasonable explanation for this finding is that as the quality of education improves, the quality of life of educated talent also improves. With more and more free mobility, it is very likely that the more educated people (with large market value) will tend to move out of the carbon-intensive areas due to the pursuit of high-quality living conditions, thereby improving the education of neighboring provinces.

### 4.8 Robustness test

This study employs the following methods to test the robustness of the model, which are shown in Table 12.

**Table 12:** Results of the robustness test.

Effect	(1)	(2)	(3)
	Replace the dependent variable	Lagging explanatory variable	Replace the spatial weight matrix
Direct	−0.723^**^ (0.300)	−0.869^***^ (0.277)	−1.061^***^ (0.274)
Spillover	−1.630^**^ (0.801)	−1.812^**^ (0.864)	−5.432^***^ (1.763)
Total	−2.353^***^ (0.854)	−2.681^***^ (0.942)	−6.494^***^ (1.822)
Control variables	YES	YES	YES
*ρ*	0.018 (0.073)	0.164^**^ (0.077)	0.263^***^ (0.098)
*N*	660	630	660
*R* ^2^	0.241	0.415	0.809
Log-L	292.822	343.400	347.192

*p < 10%, **p < 5%, and ***p < 1%.

1.Replacing the dependent variable. In addition to the CI, per capita carbon emissions (CEs) have often been adopted by researchers to measure the carbon emission level of each area. Therefore, the regression analysis is reperformed in this study by applying CEs as the dependent variable.

2.Lagging explanatory variable. Education lags behind, so it takes time to reduce the CI, and the Edu variable is lagged to test the robustness of the model.

3.Replacing the spatial weight matrix. The spillover effect and spatial agglomeration effect of Edu are affected by economic factors and geographical factors (77). Thus, the economic geographical distance weight matrix is employed to evaluate the robustness of the SDM and is expressed as follows:


$$W_{\mathrm{ij}}=0.5{W_{\mathrm{ij}}}^{G}+0.5{W_{\mathrm{ij}}}^{E},{W_{\mathrm{ij}}}^{E}=(1/|\bar{\mathrm{PGD}P_{i}}-\overset{¯|}{\mathrm{PGD}P_{j}})$$ (12)

where W_ij,_ W_ij_^G^ and W_ij_^E^ denote the economic geographical distance weight matrix, geographic distance weight matrix and economic distance weight matrix, respectively. ^−^PGDP is the average PGDP of province i from 2000 to 2021. If province i is closer to province j, W_ij_ will increase.

Columns (1)–(3) show the results of the CE, Edu_t-1_, and geographical distance spatial weight matrices, respectively. The findings show that the direction and significance of the coefficients in Tables 5, 6, 9 are consistent, revealing that the above results for the SDM are robust.

## 5 Discussion

The findings contribute to the debate on the impact of Edu on environmental variables. Unlike most studies that explore the role of industrial structure, energy consumption and technological innovation in reducing the CI (5, 6), we form a link between Edu and sustainable development, emphasising the positive role of Edu as an explanatory variable in carbon intensity reduction. The conclusions provide support for researchers who hold a “positive” view that Edu reduces the CI (3, 7, 16, 17, 25) and provide confidence to researchers who hold a “negative” view that Edu increases CEs (33, 34), possibly because Edu in those studies covers quantity dimensions rather than quality dimensions. Thus, in the long run, there is strong evidence to conclude that the Edu is a panacea for the decarbonisation of the economy beyond being merely for training in China, which further extends the human capital framework proposed by modern economic growth theory. The reasonable explanation is educational institutions are designing sustainability curricula and promoting practices for climate action to cultivate students that are capable of navigating the challenges of current and future changes in the world and meeting the SDGs and also increasing environmental awareness and lifestyle of the students and cultivating the green workforce, which can ultimately reduce CI (78). Moreover, China possesses great potential for human capital development since it ranked 43^rd^ of 122 countries in terms of the 2020 human capital index and became an ageing society in 2000. These findings also affirm the guidelines of the Paris Agreement 2017, which states that education is a central priority for every country and should be improved to solve environmental problems (79). However, for many developing economies where education remains the least prioritised subject, these results might also persuade the government to invest more in education that promotes both environmentally and economically sound growth.

Interestingly, the new evidence also shows that Edu has a significantly positive spillover effect on the CI. Notably, the spillover effect of Edu on the CI is approximately 2.2 times greater than the direct effect, indicating that the former is the main channel of effect. The spatial overflow of education passes through demonstration and peer effects and the spillover of knowledge. Moreover, Edu is more significant in reducing the CI in neighbouring provinces with low CI levels than in those with high CI levels. The possible reasons are as follows: Low CI areas are mainly in developed regions (e.g., the east), where the economy has entered the stage of sustainable development and there are higher levels of human capital stock. Moreover, educated individuals are usually associated with higher green technology and less environmental pollution (76), so they can spread the ideas of environmental protection through demonstration effects and migration behaviour. This finding complements earlier studies (3, 7, 16, 17, 25). The results also suggest that achieving carbon neutrality goals requires regional collaboration, which can be realised with regional flows and EHC sharing, as well as spatial linkages of CO_2_ reduction in terms of green awareness, production modes and lifestyles. On the one hand, these results expand the application areas of spatial economics theory and provide more ideas for future analysis of the spatial spillover of Edu and environmental variables. On the other hand, the findings can help policymakers develop regional CO_2_ emission strategies more effectively by considering the spillover effects of Edu.

On the basis of researchers exploring the impact of Edu on the CI (3, 7, 16, 17, 25), we further validate Edu’s influence mechanism. The results of the mechanism analysis illustrate that moving towards sustainability requires strategies on the technical front as well as a sustainable lifestyle and awareness at the individual level. These observations are consistent with those of other studies (5, 6), but they contradict those of other studies that confirmed the rebound effect of innovation on environmental pollution (80, 81). In terms of the interaction term, the effects of CSU are greater than those of GTI. Thus, it is crucial to guide individuals to improve their green consumption awareness and practice low-carbon lifestyles through, for example, energy conservation, recycling, saving water, consumption of eco-labelled food, and the use of eco-labelled electric appliances, which cause the carbon reduction effect of upgrading the consumption structure to be more ideal. The findings not only support ecological modernisation theory and provide empirical evidence to illuminate the “black box” of their interaction but also offer specific paths for China to achieve carbon neutrality by 2060.

The study also explores the dynamic interrelationship between Edu and CI, which complements the existing research that found the causal nexus between Edu and CO_2_ emissions, but they contradict those of other studies that confirmed unidirectional causality from Edu to the ecological footprint both in the long-run and short-run (82). The results illustrate that in terms of temporal and spatial effects, environmental policies are closely related to educational policies. The findings not only support amenity theory and provide empirical evidence to illuminate the “black box” of their dynamic interrelationship. Thus, in order for countries to survive in global competition, when formulating education policies and environmental policies, it is necessary to consider the dynamic mutual influence between the two.

## 6 Conclusion and policy implications

### 6.1 Conclusion

Carbon neutrality strategies implemented in China have gained much interest worldwide. Edu is attractive as a novel type of investment and a panacea for environmental sustainability. As a result, on the basis of spatial economics theory, this study tests the spatial correlation between Edu and the CI and then investigates the spillover effects of Edu on the CI. In addition, heterogeneity tests of the carbon intensity level, transmission mechanism and dynamic interrelationship are explored.

The results show that (a) Edu and the CI exhibit significantly positive spatial autocorrelation. (b) Edu reduces the CI mainly through the spillover effect. (c) Edu’ spillover effects vary by different CI levels. The negative spillover effects in provinces with low CI are significantly greater than the direct effects are, whereas the spillover effects in provinces with high CI are negative but not significant. Furthermore, (d) Edu reduces the CI through GTI and CSU. (e) Edu and CI have a dynamic interrelationship.

The findings have great theoretical and practical implications. At the theoretical level, the findings integrate spatial economics theory into the SDG framework, testing the potential power of Edu for macro analyses in sustainability. These findings also broaden the application of spatial theory in Edu and provide more possibilities for future research on the spatial spillover of Edu and environmental variables. These interesting findings expand upon the existing findings of spatial-spillover-free, transmission-mechanism-free and dynamic-interrelationship-free. The findings empirically design paths to achieve carbon neutrality, support amenity theory and provide empirical evidence to illuminate the “black box” of their dynamic interrelationship. At the practical level, the findings inform policymakers to develop education and achieve SDG 4, make carbon reduction policies considering the spatial effects of Edu. They also help policymakers in countries worldwide develop carbon reduction policies to achieve SDG 13 towards a net-zero future and mitigate global warming.

### 6.2 Policy implications

On the basis of the above findings, we propose the following policy recommendations to help China achieve carbon neutrality.

First, local governments should increase investment in education and pay attention to educational equity, ultimately forming a sustainable education development model and increasing the human capital stock of a low-carbon economy. Specifically, local governments can establish multilevel funding input mechanisms from the government, individuals and enterprises; increase the average years of education per capita; provide equitable access to education; improve school enrolment so that education is free for low-income and poor people; promote environmental education (82); add updated and wide content regarding climate issues to textbooks; and build low-carbon disciplines and cultivate talent in low-carbon technology fields.

Second, policymakers should pay attention to the spatial spillover of Edu when adopting differentiated emission reduction policies according to local conditions. Provinces with low CI levels can strengthen cooperation and exchange of EHC and improve the spillover and diffusion effects of Edu, further promoting the spread of green environmental awareness and upgrading green production and consumption models. Moreover, provinces with high CI levels should pay more attention to Edu’s impact on the CI in the local province. Moreover, provinces with low CI levels should fully utilise the leading and overflowing functions, which drive provinces with high CI levels to build regional education resource clusters and green innovation environments, which can realise Edu regional linkages and promote regional integration and low-carbon development (78, 79).

Finally, policymakers should promote the green technology innovation and consumption structure upgrading. For example, local governments can encourage educational institutions to invest more in the research and promotion of green technologies and carry out green technology improvement actions in key industries and key products. In addition, local governments can promote the integration of green and low-carbon lifestyles into the education system; further strengthen individuals’ environmental protection knowledge and low-carbon consumption patterns, such as achieving a low-carbon diet, low-carbon residence and low-carbon travel; and give full play to the role of the low-carbon consumption behaviour of individuals in carbon reduction.

## 7 Limitations and future work

There are several limitations that future studies can address. First, future work could consider spatiotemporal spillovers at the same time and explore some nonlinearity in the edu-CI relationships. Second, future analyses can explore the effect of the Edu on the ecological footprint since some studies have shown that the CI represents a small portion of complex issues involving environmental pollution that developing countries must address (81, 82). Third, this study selects GTI and CSU as mechanism variables. Future work can explore other mechanism channels (e.g., ICT and the energy consumption structure). Finally, the data can expand to more countries.

## Data Availability

The raw data supporting the conclusions of this article will be made available by the authors, without undue reservation.
